# The IBER study: study protocol for a feasibility randomised controlled trial of Imagery Based Emotion Regulation for the treatment of anxiety in bipolar disorder

**DOI:** 10.1186/s40814-020-00628-8

**Published:** 2020-06-15

**Authors:** Craig Steel, Kim Wright, Guy Goodwin, Judit Simon, Nicola Morant, Rod Taylor, Michael Brown, Susie Jennings, Susie Hales, Emily Holmes

**Affiliations:** 1 Oxford Health NHS Foundation Trust, Oxford, UK; 2 University of Oxford, Oxford, UK; 3 Warneford Hospital, Oxford, OX3 7JX UK; 4 University of Exeter, Exeter, UK; 5 Medical University of Vienna, Vienna, Austria; 6 University College London, London, UK; 7 University of Glasgow, Glasgow, UK; 8 University of Cambridge, Cambridge, UK; 9 University of Reading, Reading, UK; 10 Karolinska Institut, Stockholm, Sweden

**Keywords:** Bipolar disorder, Anxiety, Emotion regulation, Imagery, Psychological intervention, Feasibility

## Abstract

**Background:**

Anxiety is highly prevalent in people diagnosed with bipolar disorder (BD), and can persist between acute episodes of mania and depression. Recent studies indicate that people with BD are prone to experiencing frequent, intrusive and emotional mental images which further fuel their levels of anxiety and mood instability. These intrusive emotional mental images represent a specific target for treatment for this disorder with the potential to reduce anxiety and improve mood stability. A new brief structured psychological intervention for BD called Imagery Based Emotion Regulation (IBER) has been developed, which translates experimental work in the area of imagery and emotion into a skills training programme to improve the regulation of intrusive and distressing emotional mental images in BD. A feasibility trial is required in order to assess whether a full randomised controlled trial is indicated in order to evaluate this approach.

**Methods:**

The design is a two-arm feasibility randomised controlled trial (RCT), with 1:1 randomisation stratified by trial site and minimised on medication status and anxiety severity. Participants are 60 individuals diagnosed with bipolar disorder and experiencing at least a mild level of anxiety. Sites are defined by the geographical boundaries of two National Health Service (NHS) Trusts, with recruitment from NHS teams, GP surgeries and self-referral. The intervention is up to 12 sessions of Imagery Based Emotion Regulation within 16 weeks. The comparator is NHS standard care. The primary aim is to assess the feasibility of conducting a powered multi-site RCT to evaluate effectiveness. Measures of anxiety, depression, mania, mood stability and health care use will be conducted at baseline, end of treatment and at 16-week follow-up.

**Discussion:**

This is the first feasibility trial of an imagery-based intervention for the treatment of anxiety in bipolar disorder. If the trial proves feasible, a large multi-site trial will be required.

**Trial registration:**

ISRCTN16321795. Registered on October 16, 2018. 10.1186/ISRCTN16321795

## Background

Bipolar disorder (BD) has the highest rate of suicide of all psychiatric disorders, with up to 50% attempting suicide at least once [1]. It is highly recurrent and projected to cost the UK economy £8.2 billion per annum by 2026 [2]. Most treatments (psychosocial and pharmacological) for BD target the important outcomes of depression and relapse rates. However, current National Institute for Health and Clinical Excellent (NICE) guidelines [3] state that the evidence base of psychosocial interventions for BD is mainly of low quality. Trials have produced mixed results and due to the lack of a strong evidence base, NICE currently suggest a range of options derived from the outcomes of low to moderate quality trials. These include group interventions, psychoeducation, family-focused therapy, cognitive behavioural therapy (CBT), interpersonal and social rhythm therapy and integrated cognitive and interpersonal therapy. There is, therefore, a clear need to develop innovative interventions which directly target the mechanisms underlying mood instability.

Anxiety is highly prevalent in BD, and can persist between acute episodes of mania and depression [4–6]. Anxiety within BD is associated with increased levels of suicide, relapse, higher levels of mood fluctuation and a hampered treatment response to mood stabilisers such as lithium [7–9]. Recent studies indicate that people with BD are particularly vulnerable to experiencing frequent, intrusive and emotional mental images [6, 10] which fuel anxiety and mood instability. These images are somewhat similar to the intrusive mental ‘flashbacks’ associated with posttraumatic stress disorder (PTSD). However, intrusive mental images in BD are typically associated with imagined, emotionally intense, future events or ‘flashforwards’ e.g. an image of attempting suicide (fueling anxiety), or of winning a Nobel prize (fueling elation). Neuroimaging studies indicate that imagining an event provokes a similar response within the visual cortex to experiencing the event ‘for real’. This is likely to explain findings that image-based thought (e.g. imagining jumping off a building) induces a stronger emotional reaction than verbal–based thought (e.g. thinking purely in words about the idea of jumping off a building) [11]. Patients with BD report experiencing these mental images as ‘lifelike’, hard to ignore and difficult to control when compared to people diagnosed with other mental health problems [5, 6, 10, 12]. The intrusive emotional mental images which are highly prevalent in BD therefore represent a specific target for treatment for this disorder with the potential to reduce anxiety and improve mood stability [6, 13]. Further, the recent treatment guidelines for BD from the British Association of Psychopharmacology [14] warn of the potential adverse effects of selective serotonin reuptake inhibitors in BD and state that ‘Psychological treatments potentially offer adjunctive approaches for addressing anxiety in bipolar disorder where anxiety-specific medication is counter-indicated and/or in line with a patient’s preference’ (pg.537).

The most widely adopted form of psychological therapy within the NHS is CBT. Whilst effective for a number of disorders [15], at best CBT has produced low to moderate effects in trials targeting depression and relapse prevention in BD [16, 17]. CBT requires patients to engage in a logical verbal discussion about their emotions. Therefore, it may be that the processes adopted within CBT are not best suited to tackling emotional images within BD. Given the proposal that emotional images underlie the anxiety present in BD, it is of note that that the only study of CBT for anxiety in this group did not produce positive results [18]. There is, therefore, a need to develop a psychological intervention for BD which is distinct from traditional CBT and which improves outcomes for those diagnosed with this disabling condition.

Research in the field is also limited due to an overreliance on the assessment of mood at a single timepoint. This method fails to capture the inherent mood instability in BD. The current trial adopts an innovative, yet simple, measure of mood stability to capture this outcome. The use of single mood ratings collected every day over a 28-day period has been shown to be feasible (over 95% of data captured in our case series [19]).

We have developed a new brief structured psychological intervention for BD called Imagery Based Emotion Regulation (IBER). The treatment translates our experimental work in the area of imagery and emotion into a skills training programme to improve the regulation of intrusive and distressing emotional mental images in BD. IBER also contains a positive imagery module suitable for the small minority of BD patients who may not report anxiety-related mental images [6, 10]. It is proposed that the intervention can be delivered by mental health professionals who have received training in psychological therapies.

An early version of our intervention was adapted after input from service-users, and an improved version was then evaluated within a recently published case series [19]. Results from 14 participants indicated a *Cohen*’*s* d pre-post effect size for anxiety of 1.89 along with reduced levels of depression, improved mood stability and a high level of engagement with treatment. The specific targeting of one mechanism provides a focused intervention, which requires fewer sessions than other current psychological treatments. The potential to reduce anxiety, mood instability and relapse rates within this group is of clear health benefit to patients and has potential economic benefit to the NHS. A feasibility study is required to determine whether a full trial is indicated.

## Aim and objectives

The overall aim is to assess the feasibility and acceptability of a future definitive trial to evaluate the clinical and cost-effectiveness of IBER for reducing anxiety within adults with BD. The objectives are as follows:

1. To inform the recruitment and timeline of a full trial, by establishing the number of participants identified, approached, consented and randomised within a fixed period along with the participant retention rates for follow-up assessment and completion of intervention
2. To inform the sample size estimation of a future trial
3. To refine trial procedures by establishing the acceptability of the trial process to participants including randomisation and participant-perceived relevance and burden of the outcome measures
4. To further assess the acceptability of the treatment and, based on input from trial participants and clinicians, to further refine and develop the treatment manual and the procedures for training, supervising and assessing the competence of trial therapists

## Method

### Design

A feasibility study with a two-arm randomised parallel controlled trial design: 60 participants will be allocated to standard care (SC) or Imagery Based Emotion Regulation programme plus standard care (IBER + SC).

Patients will be randomised on a 1:1 ratio with stratification by trial site and minimised on medication status (i.e. prescribed mood stabilisers) and anxiety severity (severe anxiety being a score above 14 on a measure on the Generalised Anxiety Disorder Assessment (GAD-7 [20])) to the control or intervention arm. Web-based randomisation will be carried out independently, by the Thames Valley Clinical Trials Unit (TVCTU), using randomised permuted blocks.

Assessments will be conducted at 0 (baseline, prior to randomisation), 4 (end of treatment) and 8 months (follow-up) post-randomisation through self-report questionnaires, which will be completed via an online questionnaire system or through paper questionnaires which are posted back to the research assistant or collected in person. Research assistants will be blind to group allocation.

The CONSORT (Consolidated Standards of Reporting Trials; http://www.consort-statement.org/) extension to randomised pilot and feasibility trials statement will be followed in reporting the trial [21]. Information on the protocol is detailed in the SPIRIT (Standard Protocol Items: Recommendations for Interventional Trials) figure (see Table 1 and the Checklist in the Additional file 1).

**Table 1:** The Standard Protocol Items (SPIRIT) for a feasibility study of Imagery Based Emotion Regulation to treat anxiety in bipolar disorder (the IBER study): schedule of enrolment, interventions and assessments

	Enrolment	Baseline	Allocation	Post-intervention	16-week follow-up
Timepoint**		t1		t2	t3
Enrolment					
Eligibility screen	X				
Informed consent	X				
*[List other procedures]*					
Allocation			X		
Interventions					
[*Imager Based Emotion Regulation*]			X		
[*Standard care*]			X		
Assessments					
[*28-day mood monitoring*]		X		X	X
[*Generalised Anxiety Disorder Assessment*]		X		X	X
[Quick Inventory of Depressive Symptomatology–Self Report]		X		X	X
[Self-Rating Scale for Mania]		X		X	X
[Health Economics Questionnaire]		X		X	X
[ICECAP-A]		X		X	X
[EuroQol EQ-5D-5 L]		X		X	X
[OxCAP-MH]		X		X	X

### Setting

The setting will be secondary NHS community and inpatient services, primary care and self-referral within the geographical boundaries of two NHS Trusts: Berkshire Healthcare NHS Foundation Trust and Devon Partnership Foundation Trust.

### Participants

Participants will be 60 individuals with bipolar disorder.

#### Inclusion criteria

1. DSM-5 diagnosis of bipolar disorder (I, II or otherwise specified) assessed using the Structured Clinical Interview for DSM-5 (SCID) [22].
2. Aged 18 or above
3. Score 5 or above on the GAD-7 [20]
4. Sufficient understanding of English in order to be able to engage in the study
5. To have completed all baseline assessments, and at least 23 out of the 28 daily mood monitoring measurements conducted at baseline (see below)

#### Exclusion criteria

1. Currently within an episode of mania or depression
2. Unable to provide informed consent
3. Acute suicide risk
4. DSM-5 diagnosis of substance use or alcohol use disorder, moderate or severe, assessed using the SCID [22]
5. A change in medication within 3 months prior to randomisation
6. Currently engaged in a psychological intervention

### Trial flowchart

**Fig. 1:**
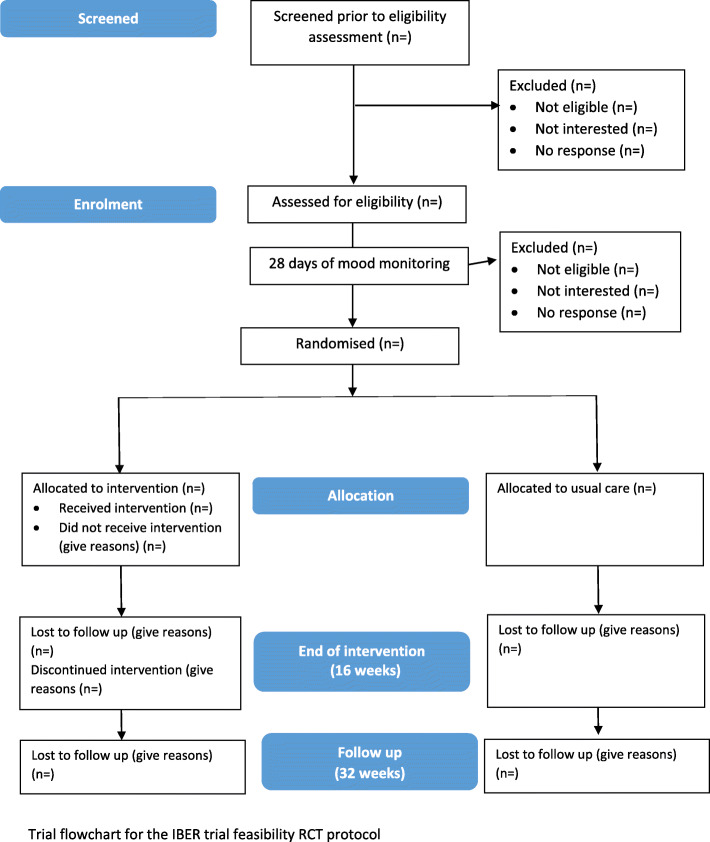
Trial flowchart for the IBER trial feasibility RCT protocol

Figure 1 illustrates the trial flowchart.

### Interventions

#### Intervention: Imagery Based Emotion Regulation (IBER)

IBER is a structured intervention delivered via 12 1-h individual sessions to be completed within 4 months. IBER involves training individuals to be able to modify and regulate their emotional reactions to intrusive mental images, and is comprised three stages:

#### Assessment

The assessment of current coping strategies and, where necessary, the development of a crisis management plan. The therapist then assesses the occurrence of intrusive mental images, including how many different images occur, how often and with what emotional impact. A personalised treatment plan is then developed in which the most significantly distressing images are identified for treatment.

#### Treatment

Four distinct theoretically informed modules have been developed as outlined below. Each distressing image is treated through training the patient in one or more of the following four techniques and strategies.

(i) Imagery rescripting (IR). Adapted from an intervention used with PTSD Participants are trained to gain control over the content of a repetitive distressing image, and therefore to be able to moderate their emotional reaction.
(ii) Visual imagery techniques (VIT). As with IR above, VIT involves techniques that enable control over the content of distressing images. These techniques are useful with a range of images, but less potent than IR for dealing with one specific repetitive image. The desired outcome is that participants learn that distressing images are not ‘real’ and are not necessarily accurate representations of their past or future.
(iii) Positive imagery. Provides skills in positive, mood-enhancing or soothing imagery which is often lacking in patients with BD. The aim is to utilise the imagery skills often present in BD to the individual’s advantage, and therefore this approach is suitable for the minority of participants who do not report experiencing intrusive anxiety-related images.
(iv) Competing tasks. Based on extensive experimental work, participants are taught to engage in certain exercises which occupy an individual’s visuo-spatial resources. Engaging in these exercises, for example the computer game ‘Tetris’, is associated with a reduction in the frequency of intrusive images. This approach is most likely to be used with the most severely distressed participants before moving onto one or more of the skills described above.

### Consolidation

The skills that have been learnt are consolidated into an action plan that the participant can draw on once the intervention is complete. The ‘visual thinking style’ associated with BD is harnessed through the development of a personal video which captures the action plan in images as well as words.

### Comparator

Both intervention and the comparator groups will receive NHS standard care (SC), likely to include the development of a risk management plan, offering lifestyle advice, including good sleeping habits and coping strategies, structured psychiatric assessment of mood, physical health and social factors and pharmacological treatment for acute episodes and long-term management.

### Treatment and intervention acceptability

Trial acceptability will be assessed via written open-ended questions about the length, time needed and acceptability of outcome measures, and views about other trial procedures. Data will be collected from all participants in both trial arms, separately from and following the 8-month assessment. Data will be analysed descriptively, with some basic content/thematic analysis of open-ended responses.

Intervention acceptability will be assessed via a service user informed semi-structured interview, conducted with 50% of the treatment arm (*n* = 15). Views of therapists delivering the intervention will be obtained via semi-structured interviews. All interviews will be audio-recorded, transcribed and analysed using thematic analysis in order to inform further possible refinements of the intervention and its delivery.

### Measures

The following measures are those proposed for use within a future definitive trial and are employed to fulfill the aims of the current feasibility study.

### Measurements in bipolar disorder

Previous studies have relied on the assessment of mood taken at one timepoint. This approach fails to capture the mood instability inherent in BD and is prone to error.

Outcomes include anxiety (primary outcome), depression and mania (secondary outcomes). Each of these is measured using self-report questionnaires covering the previous 7 days. Each symptom is measured by administering questionnaires on four separate occasions, 1 week apart, covering a 28-day period. The mean value will be used. Baseline data will cover the 28 days prior to randomisation, and follow-up data will cover the 28 days after each follow-up assessment due date (at 4 months and 8 months post randomisation).

Mood stability is measured through participants rating (0–6) how anxious, elated, sad and angry they feel on a daily basis over the same 28-day period at baseline, 4-month follow-up and 8-month follow-up. Data analysis produces a measure of mood stability for each of the four emotions, and a combined mood stability score.

### Main outcome

Anxiety: GAD-7 [20], a validated 7-item self-report measure of anxiety is widely used within NHS services.

### Secondary outcomes

Depression: Quick Inventory of Depressive Symptomatology–Self Report (QIDS-SR) [23], a validated 16-item measure of depression

Mania: Altman Self-Rating Scale for Mania (ASRM) [24], a validated 5-item self-report questionnaire for detecting mania within BD patients

Mood stability: measured over a 28-day period through the response to four mood questions (anxious, elated, sad and angry; scale 0–6) via online or paper assessments. Mood variability is quantified using the standard deviation, Teager-Kaiser energy operator and the root mean squared successive differences for each of the four daily mood measure items, and for combined mood.

Resource use and costs*:* the Health Economics Questionnaire (HEQ), [25] a comprehensive patient self-completed health economics questionnaire measuring health and social care resource use, medication, informal care, presence and absenteeism from work as well as socio-demographic background information

Quality of life*:* the EuroQol EQ-5D-5 L [26], a widely used generic health-related quality of life measure, and two broader well-being questionnaires, the Icepop Capability measure for Adults (ICECAP-A) [27] and the Oxford Capabilities questionnaire-Mental Health (OxCAP-MH) [28], two outcome instruments based on Sen’s capability approach but with different conceptual profiles, one more generic, the other one developed specifically for mental health outcome measurement

### Procedure

Participants will be identified through NHS teams, general practitioner surgeries and self-referral. For NHS teams, research assistants will discuss the trial with NHS staff who will in turn provide information on the trial to those patients who may be eligible. Patients who consent to being approached by the research team to discuss the trial further, will be provided with more information. GP surgeries within the geographical boundaries of the two participating NHS Trusts will be asked to conduct a search and post information on the trial to those potentially eligible on their patient lists. We will also provide self-referral posters within the Trusts as well as promote the trial upon relevant websites, such as Bipolar UK. The point of contact for all referrals and self-referrals will be the research assistants, who will be trained in Good Clinical Practice, and go through the participant information sheet and obtain informed consent from those who are interested. This will be followed by completing the assessments which will determine eligibility, including the GAD-7 [20] in which a minimum score of 5 (mild anxiety) is required, followed by the SCID [22] to assess for a current diagnosis of bipolar disorder, along with recording information of any other anxiety or depressive disorders, and the demographic information. Once eligibility is established, the participant completes the baseline assessment measures using the online data system adopted within the current trial. This system is used to capture all the data from the main and secondary outcome measures of the trial. In the 28 days after consent, participants are to complete daily mood ratings every day for 28 days. If an individual fails to complete at least 23 of these 28 daily ratings then they are excluded from the trial prior to randomisation. They are also to complete measures of anxiety, depression and mania every week within this 28-day period. On the final day of the 28-day assessment period, randomisation to treatment condition takes place. The participant is then randomised to receive 16 weeks of either IBER or the comparator. A member of the study team will inform the participant of the outcome by telephone. At the end of this period, another 28-day mood monitoring period occurs including the same daily and weekly measures as during the pre-randomisation baseline period. The start of this 28-day period is also the start of a 16-week period of post-intervention follow-up. At week 12 within the follow-up period, another (and final) 28-day period of daily and weekly mood monitoring begins, so as the final day of mood monitoring coincides with the end of the 16-week follow-up period. Health economic data captured once every 28 days from the start of the trial, and throughout until the end of the follow-up. Participants who do not wish to use the online data system will be provided with paper copies of the assessments to use throughout. Research assistants will remain blind to treatment allocation throughout the trial, through using separate records, room bookings and offices to the trial therapists.

After trial completion all participants, including those who dropped out of the main study, from both conditions will be posted a short questionnaire to assess their experiences of both the trial procedures (e.g. the mood monitoring assessments) and the IBER intervention (for those who received it). Also, a random 50% of those allocated to received IBER intervention will be invited to participate in a longer in-depth interview to extract qualitative data from their experience.

### Data analysis and presentation

We will not report significance tests as the feasibility RCT was not designed or powered to test hypotheses or to detect change. Given the feasibility objectives of this study, the focus of data analysis will be descriptive.

1. Objective 1 (informing recruitment and testing timeline) will be considered by summarising participant flow across both sites, reporting mean recruitment and attrition rates (both intervention and study dropouts) with 95% confidence intervals. The diagram will reflect the number of patients approached, number consenting, number randomised, number completing the intervention (defined as attending at least 50% of sessions) and number who completed the research outcome measures alongside means and standard deviations regarding the number, length and frequency of sessions. All protocol deviations, along with reasons and number of missing items on questionnaires will be reported.
2. To address objective 2 (informing future sample size estimation), mean and standard deviations of the main outcome (anxiety) for both study arms will be reported at baseline, four and 8 months.
3. To address objective 3 (acceptability of trial process and measures), descriptive statistics will be produced for quantitative acceptability data, whilst qualitative data will be subject to thematic analysis.
4. To address objective 4 (acceptability of treatment, refine treatment manual and procedures for training, supervising and assessing therapist competence), themes pertaining to treatment acceptability from the exit interviews will be described, and inter-rater reliability for the proposed competency and fidelity measures will be reported.

Feasibility for a full trial will be based on the following criteria:

1. No serious negative consequences are associated with trial participation.
2. Any concerns over the feasibility and acceptability of a full trial can be rectified.
3. Overall recruitment at 80% or above within the 12-month recruitment period
4. Eight-month follow-up data is obtained from at least 80% of participants.
5. At least 80% of participants allocated to the intervention group do not drop out (i.e. attend at least 50% of the possible sessions).

### Data management and security

Data confidentiality and secure storage will be ensured, in line with the General Data Protection Regulation 2016/679. All personal data will be kept separately from study data, so that study data will be anonymous. Participants will be identified through a trial ID number. Personal data will be kept stored in line with the NHS Code of Confidentiality. Therapy notes will be stored in line with NHS Trust policy. The research data will be held within a secure database, which will be password protected this ensuring access is only available to members of the research team. All audio-recordings will be named as the unique participant identifier and stored as computer files on secure NHS servers in an anonymised and encrypted form.

### Data quality

Data quality will be ensured through close checking and routine auditing. Given that it is anticipated that the vast majority of participants will provide data via an online system, the data obtained will be directly transferred to a database for analysis. Data collected on paper will be double checked after entry into the database.

### Study governance

Project management will be organised at a number of levels, with a part-time trial manager working alongside the chief investigator (CI), who will have overall responsibility for trial data.

### Team management

The CI will chair full team meetings (fortnightly for the first 6 months moving to monthly) to discuss all trial management issues. On alternate meetings, two external service users will be invited so as to contribute from an advisory perspective.

### Local management

Each recruitment site will hold meetings (fortnightly for the first 6 months moving to monthly) chaired by the site lead (CS/KW) and involving the research assistant (RA), service-user RA and local service/ R&D representatives to discuss recruitment and other trial activity.

Trial Steering Committee (TSC) will meet every 6 months and consist of three independent members, one of whom will be the chair, the CI, and all PIs and an invited observer representative.

Data Monitoring and Ethics Committee (DMEC) with an independent chair will review adverse events and monitor data.

The sponsor and REC will be provided with direct access to source data and other documents if required for trial review. The trial may be prematurely discontinued by the sponsor, chief investigator on the basis of new safety information or for other reasons by the DMEC.

### Dissemination

We will publish outcomes in peer-reviewed journals (open-access) and make our data available to the research community via Open Science framework. We will publish relevant data and outcomes in journals aimed at psychological therapists, service user researchers and health economists. We will file a full report in the National Institute of Health Research journals library. Team members will present at conferences to access the wide range of audiences associated with the treatment of bipolar disorder, including psychiatrists, psychological therapists, health care commissioners, charities and self-help forums. We will also disseminate via social media.

## Discussion

There is a need for innovation in the development of psychological interventions for bipolar disorder. Interventions which target specific mechanisms underlying the mood stability inherent within bipolar disorder form the basis of potentially effective new treatments. The current feasibility trial is the first to explore the potential of an imagery-based intervention for the treatment of anxiety within this disabling condition. The results will inform the development of a fully powered RCT as well as facilitate the development of training materials and a final manual of the intervention.

### Trial status

Recruitment of participants commenced in October 2018 and will be open until January 2020. Current protocol is v5.0 1.12.2018.

## Supplementary information

**Additional file 1:.** SPIRIT 2013 Checklist: Recommended items to address in a clinical trial protocol and related documents*

## Data Availability

Not currently applicable. The datasets generated and/or analysed during the current study will be available from the corresponding author on reasonable request following the publication of results.

## Abbreviations

ASRM: Altman Self Rated Mania Scale
BD: Bipolar disorder
CBT: Cognitive behaviour therapy
CI: Chief investigator
DMEC: Data Monitoring and Ethics Committee
DSM: Diagnostic and Statistical Manual
GAD: Generalised Anxiety Disorder Assessment
IBER: Imagery Based Emotion Regulation
IR: Imagery rescripting
ISRCTN: International Standard Randomised Controlled Trials Number
NHS: National Health Service
NICE: National Institute for Health and Clinical Excellence
*OxCAP-MH*: Oxford CAPabilities questionnaire-Mental Health
ICECAP-A: CEpop CAPability measure for Adults
PTSD: Posttraumatic stress disorder
QIDS-SR: Quick Inventory of Depressive Symptomatology–Self Report
RA: Research assistant
RCT: Randomised controlled trial
REC: Research Ethics Committee
SC: Standard care
SCID: Structured Clinical Interview for DSM-5
SSRI: Selective serotonin reuptake inhibitor
TSC: Trial Steering Committee
TVCTU: Thames Valley Clinical Trails Unit
VIT: Visual imagery task
